# Effect of cooking and storage temperature on resistant starch in commonly consumed Indian wheat products and its effect upon blood glucose level

**DOI:** 10.3389/fnut.2023.1284487

**Published:** 2023-11-28

**Authors:** Prabhjot Kaur, Harpreet Kaur, Renuka Aggarwal, Kiran Bains, Amrit Kaur Mahal, O. P Gupta, Lachhman Das Singla, Kulvinder Singh

**Affiliations:** ^1^Department of Food and Nutrition, Punjab Agricultural University, Ludhiana, India; ^2^Department of Mathematics, Statistics and Physics, Punjab Agricultural University, Ludhiana, India; ^3^ICAR-Indian Institute of Wheat and Barley Research, Karnal, India; ^4^Department of Parasitology, Guru Angad Dev Veterinary and Animal Sciences University, Ludhiana, India; ^5^Department of Livestock Production Management, Guru Angad Dev Veterinary and Animal Sciences University, Ludhiana, India

**Keywords:** resistant starch, glycemic index, dietary fibre, amylose, amylopectin, wheat products, cooking methods, storage temperature

## Abstract

**Background/objectives:**

The health benefits provided by resistant starch have been well documented; however, few studies are available on the resistant starch content of wheat products in India. Moreover, few studies have examined the *in vivo* efficacy of resistant starch in wheat products in improving glucose levels. This study was conducted to evaluate the effect of cooking and storage temperature on the formation of resistant starch in Indian wheat products and its effect on blood glucose levels in humans and rats.

**Methods:**

Wheat products were prepared by common cooking methods including roasting (*Chapati*), boiling (*Dalia*), Shallow frying (*Paratha*), and Deep frying (*Poori*). They were then stored at different temperatures including freshly prepared within 1 h (T1), stored for 24 h at room temperature (20-22°C) (T2), kept at 4°C for 24 h (T3) and reheated after storing at 4°C for 24 h (T4). The products were then analyzed for proximate composition (moisture, crude protein, crude fat, ash crude fibre, and carbohydrates). The effect of different cooking methods and storage temperatures on Resistant, non-resistant and total starch, total dietary fibre (soluble and insoluble), *in vitro* starch digestion rate (rapidly and slowly digestible starch), amylose and amylopectin content were analysed using standard operating procedures. The effect of products found to have higher resistant starch was studied on the post prandial blood glucose response of 10 healthy individuals using change in by analysing their glycemic index and glycemic load of wheat products. Further, the effect of resistant starch rich *chapati* on the blood glucose level of rats was also studied. Tukey’s test in factorial CRD was used to assess the effect of cooking and temperature on various parameters.

**Results:**

The amount of resistant starch was found to be high in *dalia* (boiling, 7.74%), followed by *parantha* (shallow frying, 4.94%), *chapati* (roasting, 2.77%) and *poori* (deep frying 2.47%). Under different storage temperatures, it was found high in products stored at 4°C (T3), followed by products stored at room temperature (T2), reheated products (T4) and lesser in freshly prepared products (T1). The glycemic index and glycemic load were found low in *chapati* (43, 32.3) and *dalia* (41.1, 28.6) stored at 4°C (T3) compared to others. The resistant starch content found in *chapati* stored at T3 was found to be more effective at reducing blood glucose levels in rats from 291.0 mg/100 mL to 225.2 mg/100 mL in 28 days of study compared to freshly prepared *chapati* (T1) and stored at room temperature (T2).

**Conclusion:**

Cooking methods including boiling, roasting and shallow frying increased the amount of resistant starch in foods, but cooking methods such as deep frying decreased the amount of resistant starch in food. Products stored at 4°C and at room temperature for 24 h increased the amount of resistant starch whereas the products that were freshly cooked and reheated decreased the amount of resistant starch in foods. At 4°C the stored products have a high amount of insoluble dietary fibre, slowly digestible starch, high amylose and low glycemic index. They take time to digest, meaning that they slowly increase blood glucose levels. The effect of insoluble dietary fibre and resistant starch in the inhibition of glucose diffusion in the small intestine is suggested to be due to the absorption or inclusion of the smaller sugar molecules. *In vivo* research showed that fibre and resistant starch in the digestive system of rats acts as the main factors in slowing glucose absorption and reducing a rise in blood glucose levels by promoting glycogen synthesis and inhibition of gluconeogenesis.

## 1 Introduction

Starch is a form of polysaccharide that occurs in plants with ample storage and is considered to be the most important part of a human diet. The starch present in the food is indigestible and the duration of cooking improves the digestibility of starch. Starch is made up of amylose and amylopectin chains. Amylose contains α-(1–4)-linked glucan in a straight chain, while Amylopectin contains α-(1–4) and α-(1–6)-glycosidic linkages, which results in a highly branched structure. Starch is also classified into 3 forms depending on their digestibility: rapidly digesting starch (RDS), slowly digesting starch (SDS), and resistant starch (RS). RDS is defined as a type of starch that is rapidly (within 20 min) converted into glucose molecules by enzymatic digestion. SDS is defined as a type of starch that is converted into glucose after 120 min of enzymatic digestion. (1)Whereas RS is not digested even after 120 min and it directly goes into the large intestine where it is fermented into short chain fatty acids by gut microflora (2). This can be because the digestibility of starch fraction is affected by its structure, and digestive enzymes do not hydrolyze different forms of starch structure equally (3, 4). Processing techniques may change granular starch to non granular forms (5).

Resistant starch skips digestion in the small intestine and enters directly into the large intestine. It is then fermented into short chain fatty acids (SCFAs) (6), like acetate, propionate and butyrate along with gases like H2, CO2, and CH4 by probiotic bacteria present in the large intestine (17).

There are five forms of resistant starch present in foods. Resistant starch 1 (RS1) is starch that is not accessible to digestion owing to the presence of complete, undamaged cell walls in the grains, tubers and seeds (7). Resistant starch (RS2) is a native uncooked starch granule poorly influenced by hydrolysis due to its crystalline nature. The third form of resistant starch (RS3) is retrograded starch, formed during cooking and then kept under room or low temperature (8). The fourth type of resistant starches (RS4) are those that are customized chemically to acquire resistance from digestion enzymatically such as esters and ethers of starch, and cross-linked starches (9). Fifth, resistant starch (RS5) is formed when amylose comes in contact with lipids known as amylose-lipid complexes. In many plant sources which contain a high amylose content, amylose chains are perforated by lipids and form amylose-lipid complexes (10).

The third type of starch (RS3), retrograded starch, is affected by the cooking technique used. Retrogradation is the process that causes the recrystallization of the starch chains after the gelatinized paste cools. Starch structures that are molecular or crystalline are affected by storage conditions like duration, temperature and water content, which determine the retrogradation rate and its extent. Wheat starches show higher retrogradation rates due to longer amylopectin chains and high amylose (21.7%) content compared to rice (17.55%) (11). A high amylose content leads to low digestibility in food products (12). Maize starches with high amylose content and longer chains structured themselves into double helices which resists digestion (13). Due to internal structure and B-type crystallinity, high amylose starch resists digestion by enzymes (14).

The process of starch degradation leads to the formation of starch and its by-products, which are not assimilated and absorbed in the small intestine of healthy individuals (15). Both the rate and extent of hydrolysis of starch in the small intestine determine the formation of starch by-products that play a crucial role in the body. The metabolism of resistant starch happens 5–7 h after eating as compared to typically cooked starch, which is immediately digested. Reduction in insulinemia and postprandial blood glucose occur due to the 5–7 h delay in digestion, meaning it has the potential to increase the satiety period (16). This outstanding nutritional activity, compared to dietary fibre, is mainly associated with its physiological effects. Gut-related microbiota and immune modulation, which lead to the significant production of short chain fatty acids (SCFAs), occur with normal consumption of subclasses of fermentable dietary fibre sources in the daily diet (10).

The ability of any food to raise the blood glucose level after being consumed depends on its glycemic response or glycemic index. Food that has a high glycemic index raises blood glucose levels quickly compared to low glycemic food, which slowly increases the blood glucose level. Hence, low glycemic foods are beneficial for controlling glycemic responses. Wheat, rice and maize food products have a high glycemic index and can easily raise blood glucose levels. The overconsumption of high glycemic food for longer periods can cause several metabolic disorders such as obesity and type-2 diabetes. Insulin resistance and insulin insensitivity in muscles leads to hyperinsulinemia caused by obesity (18).

Some studies have suggested that retrograded starch may reduce serum cholesterol concentration through numerous mechanisms, along with an increase in faecal bile acid excretion (19). It has been suggested that resistant starch has properties like an ability to reduce insulinemic response, postprandial glycemic responses, enhance whole body insulin sensitivity, extend satiety, and limit fat storage, and the fact that it lowers plasma cholesterol and triglyceride concentrations are exhibited by resistant starch. Thus, it could be used to prevent illnesses associated with dyslipidemia, the development of weight loss diets, and insulin resistance, and could be a dietary treatment for type 2 diabetes and coronary heart diseases (20).

The main challenge of using resistant starch in the food industry is the process of manufacturing consumer-friendly foods that contain enough resistant starch to result in the significant enhancement of public health. In response to the potential health benefits of resistant starch, the present study was undertaken to determine the effect of cooking and storage temperature on the resistant starch of wheat products that are part of the staple diet of North Indian people. We also studied the *in vivo* efficacy of resistant starch in wheat products to improve blood glucose levels.

## 2 Materials and methods

### 2.1 Procurement and the cooking process

**Figure 1:**
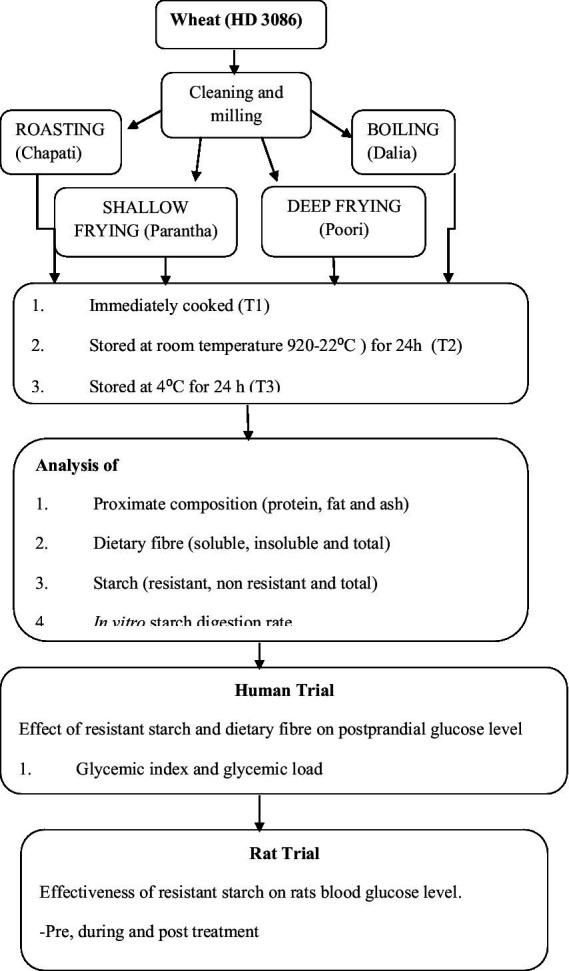
Methodology of the study.

The most commonly consumed Indian wheat variety (HD3086) was procured from the Department of Plant Breeding and Genetics, Punjab Agricultural University, Ludhiana. The grains were cleaned and ground using a sample milled with 60 mesh size for making flour and 22 mesh size for making *dalia* (Figure 1). We chose four common cooking methods: roasting, boiling, shallow frying and deep frying, which are commonly used in North Indian cuisine. We then prepared commonly consumed food products using these methods ie. *Chapat*i (flattened bread, roasting), *dalia* (broken wheat, boiling), *parantha* (shallow fried flattened bread), and *poori* (fried bread, deep frying) (Table 1). These four food products were analysed at four different conditions of storage, which were considered to be four different treatments, including freshly prepared within 1 h (T_1_) stored for 24 h at room temperature (20-22°C and 45–50% RH) (T_2_), stored at 4°C for 24 h (T_3_), and finally, reheated after being stored at 4°C for 24 h (T_4_) (Figure 1). For the study, out of five sets of each wheat product (in triplicate), one set was kept as a control (raw samples of wheat flour without any treatment) in triplicate. The other four sets (each in triplicate) were kept for cooking. After the treatments, the samples were dried and used for nutritional analysis.

**Table 1:** Preparation of commonly consumed wheat products in India.

Cereal product	Ratio of ingredients	Method
*Chapat*i (flattened bread, roasting)	Whole wheat flour to water (2·5: 1, w/v)	Whole wheat flour was kneaded with the addition of water and made into a soft dough. About 25 g of dough was taken and flattened into a *chapati* by using a wooden rolling pin and board to 10–12 cm diameter and then toasted for 2 min directly on a hot iron tawa without smearing oil on both sides till golden brown.
*Dalia* (broken wheat, boiling)	Broken wheat and water (1–7.5 w/v)	*Dalia* was prepared by mixing broken wheat and water and then boiling it for 10 min in an open pan.
*Parantha* (shallow fried flattened bread)	Whole wheat flour and water (2·5: 1, w/v)	Whole wheat flour was kneaded by the addition of water and made into a soft dough. About 30 g of dough was taken and flattened using a wooden rolling pin and board to 10–12 cm diameter and then about 1.5 mL of oil was smeared and the *parantha* was folded 4 times and again flattened and put on hot tawa and toasted for 2 min on both sides with smeared oil until golden brown in color.
*Poori* (fried bread, deep frying)	Whole wheat flour and water (2.5:1, w/v)	The whole wheat flour, a pinch of salt and water were mixed and left to settle for 10 min. Approximately 20 g of dough was taken and flattened and then deep fried in mustard oil (preheated at a temperature of 200–220°C to prevent the food from sticking) for 35–40 s until golden-brown in colour, then removed from the oil.

### 2.2 Nutritional analysis

Nutritional analyses of raw and cooked samples were undertaken, examining crude protein, crude Fat and ash using standardized methods. The macro-Kjeldahl method was used for the determination of crude protein. Crude fat and ash content were also measured by using (AOAC 2000) method (21).

#### 2.2.1 Dietary fibre

The total dietary fibre was determined using a megazyme total dietary fibre (K-TDFR-200A) kit. The soluble and insoluble dietary fibre contents were also analyzed using the standard protocol given by (21). The dietary fibre was calculated using the formula:


$$\text{Dietary fiber (\%)}=\frac{R+1R‐2p‐A‐B}{2}\times 100$$

$$\frac{m+1m2}{2}$$

Where: R_1_ = residue weight 1 from m1, R_2_ = residue weight 2 from m_2_, m_1_ = sample weight 1, m_2_ = sample weight 2, A = ash weight from R_1_, p = protein weight from R_2_ and B = blank Where: BR = blank residue, BP = blank protein from BR1, BA = blank ash from BR2.

#### 2.2.2 Total starch and resistant starch

The total starch and resistant starch were determined using a megazyme K-RSTAR assay, as outlined previosuly (22). Resistant starch and non-resistant (solubilized) starch were added to determine the total amount of starch.

#### 2.2.3 *In vitro* starch digestion rate

The *in vitro* starch digestion rate was determined using the procedure given in another study (23). In total, 500 mg of the sample was exposed for 15–20 s to 250 U porcine amylase in 1 mL of synthetic saliva (carbonate buffer; Sigma A-3176 Type VI-B). Then, 5 mL of pepsin (1 mL per ml of 0.02 M aq. HCl; from gastric porcine mucosa; Sigma P-6887) was added and incubated for 30 min in a water bath at 37°C. The digesta was neutralized by adding 0.02 M aq. Sodium hydroxide (5 mL) before adjusting the pH 6. (25 mL of 0.2 M C2H3NaO2 buffer) 5 mL of amyloglucosidase (Sigma A-7420 from Aspergillus niger; 28 U per mL of acetate buffer) and pancreatin (2 mg per mL of acetate buffer; Sigma P1750 from porcine pancreas) were added. Then the solution was incubated for 4 h, and at various times during that period, an Accucheck glucometer was used to monitor the digesta’s glucose concentration.

#### 2.2.4 Rapidly digestible starch and slowly digestible starch

The glucometer reading at 15 min was converted to the percentage of starch digested using the following equation


$$\text{Where: }DS=\frac{0.9\times G\times G180\times V}{W\times S\left[100-M\right]}$$

GG = Reading of the glucometer (mM/L). V = Digest volume (mL), 180 = glucose’s molecular weight W = sample weight (g). S = sample’s starch content (g per 100 g dry sample). M = moisture percentage in the sample (g per 100 g sample). 0.9 = starch stoichiometric constant from glucose concentrations.RDS% = percentage of starch digested at 15 min. SDS% = percentage of starch digested at 120 min – percentage of starch digested at 15 min.

#### 2.2.5 Amylose and amylopectin

The Amylose Content was measured by colorimetric estimation of the amylose-iodine complex (24). The defatted sample weighing 100 mg was taken in a boiling tube and mixed with 1 mL of distilled ethanol. Then, 9 mL of 1 N sodium hydroxide was added and the tube was placed in a boiling water bath for 10 min. The volume was made up to 100 mL, out of which 5 mL were transferred to a 100 mL volumetric flask, mixed with 1 mL 1 N acetic acid (MP Biomedicals) and 2 mL iodine solution (1 g iodine and 10 g KI/500 mL distilled water) and kept in darkness for 20 min. Finally, the volume was made to 100 mL and the absorbance was measured at 620 nm using a blank 5 mL 0.09 N NaOH, to which acetic acid (1 mL) and iodine solution (2 mL) were added in 100 mL total volume.

Amylopectin = 100-amylose.

### 2.3 Impact of resistant starch and soluble fibre components on postprandial glucose response by measuring glycemic index

The glycemic index was calculated using the method given by Goni (25). Ten healthy individuals were selected for measurement of blood glucose levels. The food was given in the morning after 12 h of fasting and the food was eaten within 15 min. Blood samples were taken using a finger-prick using a Glucometer (Dr. Morphine). Blood glucose levels were measured at fasting 0, 15, 30, 45, 60, 90, and 120 min after taking 50 g of carbohydrates in the form of cooked cereal products. To compare the effect of cooked food on blood glucose, the control sample was also given in the form of 50 g of glucose. Volunteers were allowed to drink 150–300 mL of water depending on the food consumed during the study. Then, the glycemic index was calculated by applying the formula,


$$GI=\frac{Areaunderthecurvefor\;50gm\;carbohydratefortestsample}{Areaunderthecurvefor\;50gm\;carbohydratesfromcontrol\;\left(glucose\right)}X\;100$$

Glycemic load was calculated as:


$$Glycemicload=\frac{GI\times Availablecarbohydrates}{100}$$

### 2.4 Effectiveness of resistant starch on blood glucose level in rats

Previous human supplementation research has indicated that cooked wheat products that had been subject to different treatments had a low glycemic index. We hypothesized that lower glycemic index foods have a positive effect on treating diabetes maybe through enhanced insulin secretion or by reducing its sensitivity. For this, we conducted a rat experiment to have authentic, real and unbiased data. Moreover, rats are very similar to humans genetically, and biologically and their behavioral characteristics closely resemble that of humans.

#### 2.4.1 Animal collection

35 Wistar albino rats aged 2–3 months with weights 180-220 g were obtained from the animal house and breeding centre (AHBC) at Akal College of Pharmacy and Technical Education Mastuana Sahib, Sangrur (Registered breeder of CCSEA). The experiment was conducted as per the permission provided by the Institutional Animal Ethics Committee (IAEC no.:- GADVASU/2023/1AEC/68/12). The animals were housed in cages, fed with commercial pellets and had access to water *ad libitum*.

#### 2.4.2 Induction of diabetes

The Wistar albino rats were given an intraperitoneal injection of freshly prepared 230 mg/kg Nicotinamide (NA) with buffer saline NaCl 0.9%. After 15 min, the rats were again given intraperitoneal injections of Streptozotocin (STZ) at about 60 mg/kg. Rats were provided with 5% of glucose water after injection to prevent hypoglycaemia. After 5 days of induction, their blood samples were taken and used to measure blood glucose and insulin levels. A blood glucose level of more than 200 mg/kg was an indicator of diabetic rats. Rats were treated for 28 days and blood glucose levels were checked first, third, and last week of the experiment.

#### 2.4.3 Treatment protocol

1. Group-I: (Normal control) consists of normal rats given a normal diet for 28 days.
2. Group-II: (Diabetic control) after induction of diabetes were given a normal diet for 28 days.
3. Group-III: (Treatment group) Diabetic rats with supplement FWC (freshly prepared wheat *chapati*, T1) orally for 28 days.
4. Group-IV: (Treatment group) Diabetic rats with supplement 24WC (24 h stored at room temperature wheat *chapati,* T2) orally for 28 days.
5. Group-V: (Treatment group) Diabetic rats with the supplement RehWC (reheated wheat *chapati* after storing 4°C for 24 h, T4) orally for 28 days.

Rats did not eat the diet in Treatment 3 as it was not accepted by the subjects due to its cold temperature. Moreover, as it is not the habit of people in North India to consume this product at this temperature, it was not fed to the rats.

## 3 Results

### 3.1 Proximate composition

The crude protein content was found to be highest in *Chapati* (10.79%), which was prepared using roasting followed by *dalia* (boiling,9.53%), *parantha* (shallow frying, 9.46%), and *poori* (deep frying, 9.16%) (Table 2). A significant difference (≤0.001*) was also observed in the protein content of the products stored at different temperatures with the highest content in T2. The protein content of *chapatti* (11.06%) *parantha* (11.72%) and *dalia* (9.22%) was found highest in T3, i.e., when stored at 4°C for 24 h. This was followed by T2, T1, and T4 in *chapatti* and T2, T4, and T1 in *parantha* and T4, T1, and T2. P*oori* stored for 24 h at room temperature (20-22°C and 45–50% RH) showed the highest protein content of 10.98% compared to other storage temperatures. All methods of cooking led to a decline in the protein content of the products as compared to the raw uncooked sample of wheat flour (11.3%).

**Table 2:** Effect of different cooking methods and storage temperatures on crude protein, crude fat, and ash content of wheat products (per 100 g).

Wheat products	Treatments				
	T1	T2	T3	T4	Treatment Mean
Crude Protein (raw/ flour)	11.36 ± 0.56				
Chapati	10.59 ± 0.09^b^	11.02 ± 0.54^b^	11.06 ± 0.07^b^	10.52 ± 0.20^b^	10.79^A^
Parantha	8.01 ± 0.22^ef^	9 ± 0.10^cd^	11.72 ± 0.25^a^	8.13 ± 0.33^de^	9.46^BC^
Poori	9.03 ± 0.17^cd^	10.98 ± 0.27^b^	9.39 ± 0.16^ef^	8.26 ± 0.46^c^	9.16^C^
Dalia	9.16 ± 0.66^c^	8.84 ± 0.37^b^	9.22 ± 0.17^f^	8.91 ± 0.32^b^	9.53^B^
Storage mean	9.21^C^	10.45^A^	9.58^B^	9.70^B^	
Crude fat (raw/flour)	1.62 ± 0.29				
Chapati	1.39 ± 0.14^efg^	1.30 ± 0.07^fg^	1.48 ± 0.03^defg^	1.08 ± 0.11^g^	1.31^C^
Parantha	2.42 ± 0.13^d^	2.35 ± 0.17^def^	2.45 ± 0.01^defg^	2.41 ± 0.08^de^	2.27^B^
Poori	12.05 ± 0.68^a^	11.56 ± 0.94^b^	12.23 ± 0.36^c^	11.26 ± 0.56^c^	13.02^A^
Dalia	1.86 ± 0.05^defg^	1.70 ± 0.06^defg^	2.30 ± 0.18^defg^	1.99 ± 0.01^defg^	1.89^B^
Storage Mean	5.43^A^	4.80^B^	4.07^C^	4.18^C^	
Ash (raw/flour)	0.83 ± 0.16				
Chapati	1.36 ± 0.05^bcd^	1.60 ± 0.05^abc^	1.87 ± 0.03^a^	1.21 ± 0.12^d^	1.51^AB^
Parantha	1.65 ± 0.14^ab^	1.58 ± 0.06^abc^	1.82 ± 0.06^a^	1.29 ± 0.06^cd^	1.58^A^
Poori	1.20 ± 0.20^d^	1.12 ± 0.07^d^	1.24 ± 0.24^d^	1.41 ± 0.08^bcd^	1.24^C^
Dalia	1.43 ± 0.13^bcd^	1.44 ± 0.09^bcd^	1.59 ± 0.06^abc^	1.33 ± 0.10^bcd^	1.44^B^
Storage mean	1.40^BC^	1.43^B^	1.62^A^	1.31^C^	

Values are mean ± SD; Mean values with different superscripts are significantly (*p* ≤ 0.05) different. T1 - freshly prepared within 1 h, T2 - stored at room temperature (20-22°C for 24 h), T3 - Kept at 4°C for 24 h, T4 – samples reheated after being stored at 4°C for 24 h.

Wheat chapati (roasting); Parantha (shallow-frying); Poori (deep-frying); Dalia (boiling).

Contrary to protein, the crude fat content was observed to be higher in *poori* (deep frying, 13.02%) followed by *parantha* (shallow frying, 2.27%) than the raw and other cooked products because of the addition of extra oil. Among different storage temperatures, crude fat content was high in T1 with 5.43%, followed by T2 (4.80%), T4 (4.18%), and T3 (4.07%). Raw wheat samples contained 0.83% ash, which was increased after cooking. The ash content was found to be high in *parantha* (shallow frying, 1.58%), followed by *chapati* (roasting, 1.51%), *dalia* (boiling, 1.44%), and *poori* (deep frying, 1.24%). Keeping the products at 4°C for 24 h (i.e., treatment T3) resulted in higher ash content, followed by the products stored at room temperature (T2).

### 3.2 Dietary fibre

The soluble (2.05%), insoluble (10.78%) and total dietary fibre (12.84%) were found to be maximum in *dalia*, which was prepared by boiling. Storing of the prepared food products at different temperatures, affected the dietary fibre content. The insoluble and total dietary fibre content increased with an increase in storage period at low temperatures and was observed to be highest in products stored at 4°C (T3) (10.43, 12.20%) for 24 h while the soluble fibre content was higher in the fresh food samples (T1) (2%) (Table 3).

**Table 3:** Effect of different cooking methods and storage temperatures on dietary fibre (Soluble, Insoluble, and Total) content of cereal products (per 100 g).

Wheat products	Treatments				
	T1	T2	T3	T4	Treatment mean
Raw/flour (soluble dietary fibre)	1.41 ± 0.02				
Chapati	1.97 ± 0.06^bcde^	1.87 ± 0.06^defg^	1.77 ± 0.06^fgh^	1.90 ± 0.00^cdef^	1.87^B^
Parantha	1.64 ± 0.06^hi^	1.33 ± 0.06^jk^	1.03 ± 0.06^l^	1.23 ± 0.06^k^	1.31^D^
Poori	2.10 ± 0.10^b^	1.83 ± 0.06^efgh^	1.47 ± 0.06^ij^	1.67 ± 0.08^gh^	1.76^C^
Dalia	2.30 ± 0.10^a^	2.03 ± 0.06^bcd^	1.83 ± 0.06^efgh^	2.07 ± 0.06^bc^	2.05^A^
Storage Mean	2.0^A^	1.76^B^	1.52^C^	1.71^B^	
Raw/flour (Insoluble dietary fibre)	9.48 ± 0.13				
Chapati	9.45 ± 0.09^i^	9.83 ± 0.06^h^	10.17 ± 0.06^fg^	9.27 ± 0.12^ij^	9.68^D^
Parantha	10.43 ± 0.12^de^	10.83 ± 0.06^b^	11.10 ± 0.10^a^	10.37 ± 0.06^def^	10.68^B^
Poori	9.10 ± 0.10^j^	10.23 ± 0.06^efg^	10.80 ± 0.10^bc^	10.07 ± 0.06^gh^	10.05^C^
Dalia	10.41 ± 0.08^de^	10.83 ± 0.06^b^	11.33 ± 0.06^a^	10.57 ± 0.06^cd^	10.78^A^
Storage Mean	9.85^D^	10.43^B^	10.85^A^	10.06^C^	
Raw/flour (Total dietary fibre)	10.89 ± 0.12				
Chapati	11.42 ± 0.03^gh^	11.70 ± 0.00^efg^	11.93 ± 0.06^de^	11.17 ± 0.12^h^	11.55^D^
Parantha	12.08 ± 0.17^cd^	12.17 ± 0.06^cd^	12.13 ± 0.15^cd^	11.60 ± 0.00^fg^	11.99^B^
Poori	11.20 ± 0.17^h^	12.07 ± 0.06^cd^	12.27 ± 0.12^c^	11.74 ± 0.10^ef^	11.81^C^
Dalia	12.71 ± 0.15^b^	12.87 ± 0.06^ab^	13.17 ± 0.06^a^	12.63 ± 0.06^b^	12.84^A^
Storage Mean	11.85^C^	12.20^B^	12.37^A^	11.78^C^	

Values are mean ± SD; Mean values with different superscripts are significantly (*p* ≤ 0.05) different. T1-freshly prepared within 1 h, T2-stored at room temperature (20-22°C for 24 h), T3-Kept at 4°C for 24 h, T4-samples reheated after being stored at 4°C for 24 h.

Wheat chapati (roasting); Parantha (shallow-frying); Poori (deep-frying); Dalia (boiling).

### 3.3 Resistant starch and total starch

The resistant starch content of the raw samples (0.52%) increased after cooking and the amount of resistant starch was found to be highest in *dalia* (boiling, 5.45%) followed by *parantha* (shallow frying, 3.46%), *chapati* (roasting, 2.37%), and *poori* (deep frying, 2.04%). Wheat products stored at T3 were found to have a higher amount of resistant starch content (4.47%) followed by T2 (3.32%), T4 (2.97%) and a lesser amount in freshly prepared products T1 (2.57%) (Table 4). On the other hand, non-resistant starch content in raw samples (70.57%) was higher, which was reduced after cooking. The content was highest in *chapati* (roasted, 68.45%) and *poori* (deep frying, 68.42%), respectively and during storage it was found to be high during T1 (68.8%) compared to T3 (66.1%) (Table 5). No significant difference was seen in the total starch content of raw and cooked samples after cooking. Only the type of starch (resistant and non significant starch) was affected. Results showed that the total starch content in wheat products lies between (69.9 to 72.93%) with the highest level of resistant starch at 72.93% in *dalia* with T1 and the lowest level at 69.9% in T3 *chapati* (Table 6).

**Table 4:** Effect of different cooking methods and storage temperatures on the resistant starch content of cereal products (per 100 g).

Wheat products	Treatments				
	T1	T2	T3	T4	Treatment Mean
Raw/ flour	0.523 ± 0.02				
Chapati	2.13 ± 0.02^ij^	2.39 ± 0.01^hi^	2.77 ± 0.02^fg^	2.21 ± 0.02^hij^	2.37^C^
Parantha	2.30 ± 0.10^hi^	3.60 ± 0.01^e^	4.94 ± 0.04^bc^	3.03 ± 0.15^f^	3.46^B^
Poori	1.73 ± 0.06^k^	2.07 ± 0.06^ij^	2.47 ± 0.06^gh^	1.90 ± 0.00^jk^	2.04^D^
Dalia	4.13 ± 0.12^d^	5.23 ± 0.21^b^	7.74 ± 0.06^a^	4.73 ± 0.29^c^	5.45^A^
Storage Mean	2.57^D^	3.32^B^	4.47^A^	2.97^C^	

Values are mean ± SD; Mean values with different superscripts are significantly (*p* ≤ 0.05) different. T1-freshly prepared within 1 h, T2-stored at room temperature (20-22°C for 24 h), T3-Kept at 4°C for 24 h, T4-samples reheated after being stored at 4°C for 24 h.

Wheat chapati (roasting); Parantha (shallow-frying); Poori (deep-frying); Dalia (boiling).

**Table 5:** Effect of different cooking methods and storage temperatures on the non resistant starch content of cereal products (per 100 g).

Wheat products	Treatments				
	T1	T2	T3	T4	Treatment Mean
Raw/flour	70.57 ± 0.40				
Chapati	69.60 ± 0.53^a^	68.13 ± 0.12^abcd^	67.13 ± 0.12^cdef^	68.93 ± 0.95^ab^	68.45^A^
Parantha	68 ± 0.85^bcde^	67 ± 0.80^def^	66.47 ± 0.67^ef^	67.03 ± 0.06^def^	67.12^B^
Poori	69.07 ± 0.67^ab^	68.63 ± 0.38^abc^	67.53 ± 0.35^bcde^	68.47 ± 0.29^abcd^	68.42^A^
Dalia	68.80 ± 0.17^ab^	65.60 ± 0.35^f^	63.47 ± 0.40^g^	66.47 ± 0.58^ef^	66.0^C^
Stoarge Mean	68.8^A^	67.3^B^	66.1^C^	67.2^B^	

Values are mean ± SD; Mean values with different superscripts are significantly (*p* ≤ 0.05) different. T1-freshly prepared within 1 h, T2-stored at room temperature (20-22°C for 24 h), T3-Kept at 4°C for 24 h, T4-Samples reheated after being stored at 4°C for 24 h.

Wheat chapati (roasting); Parantha (shallow-frying); Poori (deep-frying); Dalia (boiling).

**Table 6:** Effect of different cooking methods and storage temperatures on the total starch content of cereal products (per 100 g).

Wheat products	Treatments				
	T1	T2	T3	T4	Treatment Mean
Raw/flour	71.09 ± 0.41				
Chapati	71.73 ± 0.52^ab^	70.52 ± 0.10^bc^	69.9 ± 0.14^c^	71.15 ± 0.95^bc^	70.82^B^
Parantha	70.30 ± 0.92^bc^	70.60 ± 0.81^bc^	71.40 ± 0.71^c^	70.07 ± 0.21^c^	70.59^B^
Poori	70.80 ± 0.66^bc^	70.70 ± 0.35^bc^	70 ± 0.30^c^	70.37 ± 0.29^bc^	70.46^B^
Dalia	72.93 ± 0.06^a^	70.83 ± 0.25^bc^	71.20 ± 0.46^bc^	71.2 ± 0.50^bc^	71.5^A^
Storage Mean	71.4^A^	70.66^B^	70.62^B^	70.69^B^	

Values are mean ± SD; Mean values with different superscripts are significantly (*p* ≤ 0.05) different. T1-freshly prepared within 1 h, T2-stored at room temperature (20-22°C for 24 h), T3-Kept at 4°C for 24 h, T4-samples reheated after being stored at 4°C for 24 h.

Wheat chapati (roasting); Parantha (shallow-frying); Poori (deep-frying); Dalia (boiling).

### 3.4 *In vitro* starch digestion rate

**Figure 2:**
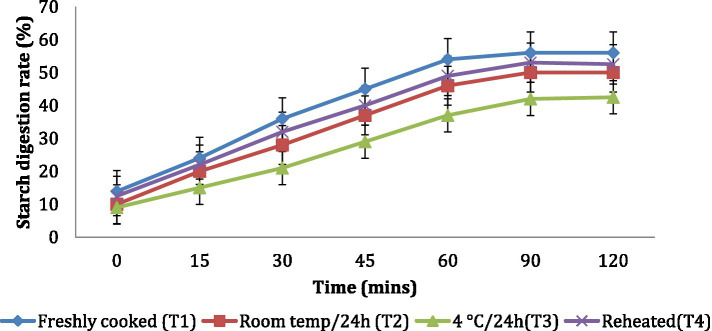
Effect of different storage temperatures on *in vitro* starch digestion rate of wheat chapati.

**Figure 3:**
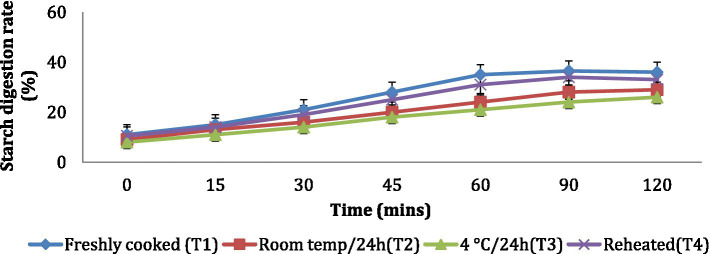
Effect of different storage temperatures on *in vitro* starch digestion rate of wheat dalia.

**Figure 4:**
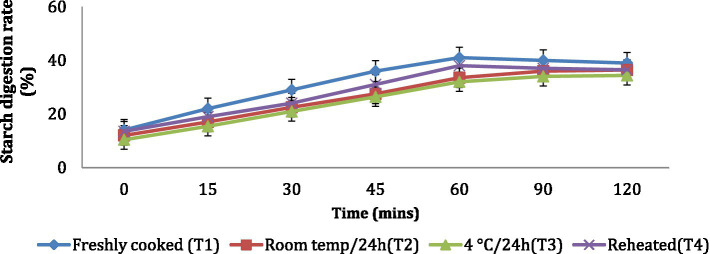
Effect of different storage temperatures on the *in vitro* starch digestion rate of wheat paratha.

**Figure 5:**
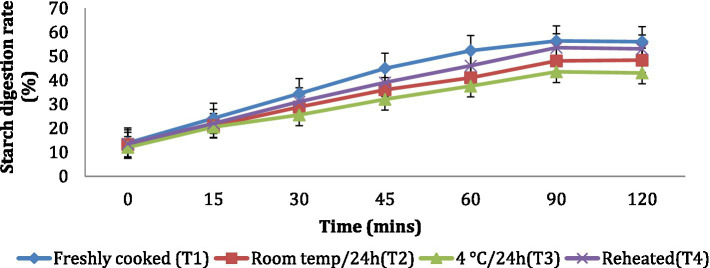
Effect of different storage temperatures on the *in vitro* starch digestion rate of wheat poori.

The *in vitro* starch digestion rate is important in determining the potential of a food product to raise the blood glucose levels of an individual. The *in vitro* starch digestion rate of wheat products (*chapati, dalia, paratha and poori*) was affected by different storage temperatures. and was determined at 120 min after completion of the digestion of the food sample (Figures 2–5). The wheat *chapati* stored with T3 and T2 had a slower digestion rate of 42.5 and 50% at 120 min compared to T1 and T4 treated *chapati* which had a completed digestion rate of 56 and 53% at 90 min. In wheat *dalia*, the rate of starch digestion was lower in T3 and T2 with 26 and 29% at 120 min as compared to T1 (36%) and T4(33%). The starch digestion rate of wheat *paratha* was high in T1 (41%) followed by T4 (38%), T2(33.5%) and T3 (32%) at 60 min. In *poori*, the starch digestion rate was also found high in T1 (56%) and T4 (53%) compared to products stored at low temperatures for 24 h (T2 and T3).

### 3.5 Rapidly digestible starch (RDS) and slowly digestible starch (SDS)

**Figure 6:**
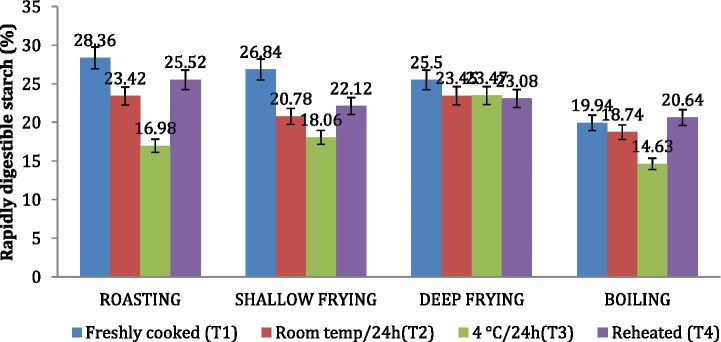
Effect of different cooking methods and storage temperatures on rapidly digestible starch in wheat products.

**Figure 7:**
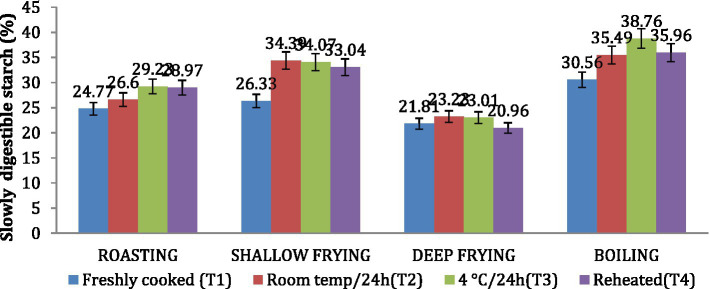
Effect of different cooking methods and storage temperatures on slowly digestible starch in wheat products.

Similar to the *in vitro* starch digestion rate, the amount of SDS in *dalia* (boiling, 38.76%) was highest followed *parantha* (shallow frying, 34.07%), *chapati* (roasting, (29.23%) and *poori* (deep frying, 23.01%). Treatment 3(T3) increased the level of SDS to the maximum followed by T2, T4 and T1. Due to higher SDS content (Figures 6, 7). *Dalia* (boiling, 14.63%) was observed to have the lowest RDS followed by *chapati* (roasting, 16.98%), *parantha* (shallow frying, 18.06%), and *poori* (deep frying, 23.47%) when stored with treatment 3.

### 3.6 Amylose and amylopectin

**Figure 8:**
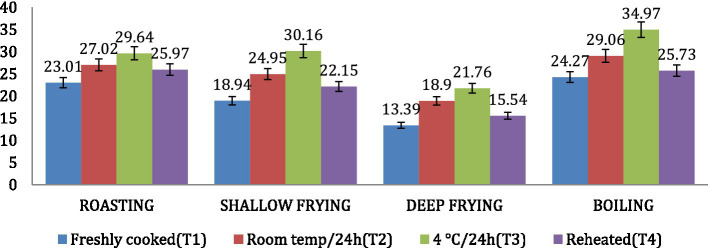
Effect of different cooking methods and storage temperatures on the amylose content in wheat products.

**Figure 9:**
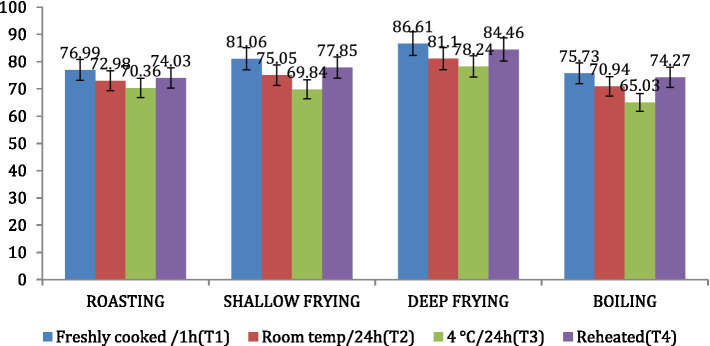
Effect of different cooking methods and storage temperatures on amylopectin content in wheat products.

The amylose content was found to be highest in *dalia* (boiling, 34.97%), followed by *parantha* (shallow frying, 30.16%), *chapati* (roasting, 29.64%) and *poori* (deep frying, 21.76%). Treatment 3 resulted in increased amylose content of the products followed by T2, T4 and T1 (Figure 8). On the other hand, the amylopectin content was found highest in *poori* (deep frying, 86.61%), followed by *parantha* (shallow frying, 81.06%), *chapati* (roasting, 76.99%) and *dalia* (boiling, 75.73%) respectively and under storage conditions, it was found highest with T1 followed by T4, T2, and T3 (Figure 9).

### 3.7 Impact of resistant starch and soluble fibre components on postprandial glucose response

Wheat products like *chapati* and *dalia* having high RS content with all the treatments were selected for feeding to 10 healthy human subjects to evaluate glycemic response. However, chapattis having treatment 3 was not accepted by the subjects due to its cold temperature so was denied by them for its consumption. Moreover, in the north of India people tend not to consume this product at this particular temperature. Therefore, treatment 3 was not considered for the evaluation of the glycemic index of the chapattis. Similarly, for the wheat dalia, we had to discard the product, having treatment 2 as storing at this temperature, led to microbial growth in the product. The lowest glycemic index was observed after the consumption of d*alia* (41.12%) with T3 followed by *chapati* (45.2%) and treatment 2 with a glycemic load of 28.6 and 35.5%, respectively. Treatments 2 and 3 were found to be the best storage conditions for lowering the glycemic index and may prove beneficial for diabetic individuals (Table 7).

**Table 7:** Effectiveness of wheat products stored at different temperatures on the glycemic Index and glycemic load of human subjects.

Wheat products	Treatments				
Glycemic Index	T1	T2	T3	T4	*p*-Value
Wheat chapati	54.18 ± 1.45^a^	45.2 ± 2.2^c^	-	51.0 ± 1.19^b^	≤0.001*
Dalia	50.3 ± 1.5^a^	-	41.1 ± 1.3^c^	47.5 ± 1.3^b^	≤0.001*
Glycemic Load					
Wheat chapati	39.7 ± 1.06^a^	35.5 ± .66^b^	-	38.4 ± .90^a^	≤0.001*
Dalia	34.9 ± 1.07^a^	-	28.6 ± .96^c^	32.5 ± .93^b^	≤0.001*

Values are mean ± SD of 10 subjects; Different small letters in different rows show significant differences at 5%; *significant at 5%; NS, non-significant. T1-freshly prepared within 1 h, T2-stored at room temperature (20-22°C for 24 h), T3-Kept at 4°C for 24 h, T4-samples reheated after being stored at 4°C for 24 h.

### 3.8 Effectiveness of resistant starch on blood glucose level in rats

Similar to human experiments, treatment 3 for *chapatis* was not considered for the rat trial. *Dalia* was not given to rats due to the limited number available to us and because *dalia* is consumed once or twice a week whereas *chapatis* tend to be consumed thrice a day in north India. The results indicated that resistant starch from different diet groups had a decreasing tendency in blood glucose concentrations, but the mean values were not different from either control or diabetic control groups. Blood glucose levels were significantly increased from 114.3 ± 6.9 in normal rats to 286.5 ± 16.5 mg/ 100 mL in diabetic rats. However, significantly, the levels in pre-treatment groups returned to near normal range from each treatment group (G3-G5) after giving the treatment Diet. In G3, *chapati* stored at T1 was given and blood glucose level reduced from 275.8 ± 29 to 225.5 ± 20.0. In G4, *chapati* stored at T2 was given and found blood glucose level reduced from 291.0 ± 9.8 to 225.1 ± 15.0. In G5 *chapati* stored at T4 was given and found blood glucose levels reduced from (292.0 ± 12.1 to 256.3 ± 12.4), respectively (Table 8). The diet results in G3, G4, and G5 found 81.7, 77.3, and 87.7% decreases in the blood glucose levels in the rats.

**Table 8:** Effectiveness of resistant starch on blood glucose level in rats.

Diet group	Pre treatment	During treatment	Post treatment	Treatment Mean
G1	114.3 ± 6.86^g^	115 ± 5.4^g^	112.6 ± 4.32^g^	114.0^E^
G2	286.5 ± 16.5^a^	285.6 ± 13.8^a^	283.3 ± 8.35^a^	285.1^A^
G3	275.8 ± 29^abc^	259.3 ± 28.5^abcde^	225.5 ± 20.0^ef^	256.8^BC^
G4	291.0 ± 9.8^a^	269.3 ± 14.0^abcd^	225.2 ± 15.0^f^	255.1^BC^
G5	292.0 ± 12.1^a^	279.17 ± 14.3^ab^	256.3 ± 12.4^abcde^	275.8^AB^

Each value is the mean of six observations, Values are mean ± SD; Mean values with different superscripts are significantly (*p* ≤ 0.05) different. Table shows the standard of the mean of diet groups; 1. Control group (normal diet fed rats) (G1); 2. Diabetic control (Normal diet fed rats) (G2); 3. Freshly prepared within 1 h wheat chapati fed rats (G3); 4. Wheat chapati stored at room temperature (20-22°C) for 24 h fed rats (G4); 5. Samples reheated after being stored at 4°C for 24 h storage fed rats.

## 4 Discussion

The present study aimed to find the best storage conditions and cooking methods for increasing the resistant starch content of wheat products commonly consumed in India. The protein content was found to be higher in raw flour (without any treatment) compared to cooked examples, as cooking leads to denaturation of the protein structure. The milling method and the mesh size used in the current study might have also had an effect on the nutritional composition of the flour samples. The protein content of all wheat products was found to be low in freshly prepared products, i.e., within 1 h (T_1_) and the products were reheated after being stored at 4°C for 24 h (T_4_).

Food stored at low temperatures and room temperature had a higher amount of moisture content compared to freshly prepared and reheated products. This might be because protein content increased with the increase in the moisture content of food (26). In a study, protein content was found to decrease with increasing temperature from 4 to 25°C and the greatest increase in the protein content of quinoa (Titicaca) was found to happen in storage conditions of 4°C (3.35% increase) and 10°C (3.71% increase) respectively (27). Similarly in another study, the soluble protein content in steamed bread was increased up to 2.03 mg/g when frozen at-12°C. During the freezing process, the soluble protein contents increased from 1.57 mg/g to 1.75 mg/g at-24°C. This might be due to the fact that storing food at low temperatures redistributes water and ice recrystallization, which destroys the internal structure of gluten proteins and leads to depolymerization and shedding of gluten and an increase in protein solubility (28).

Fat is the prerequisite of every food as it increases the palatability of the product. In the roasting and boiling techniques used for the preparation of products, fat content was not changed significantly compared to raw uncooked samples, but in shallow and deep frying methods, additional fat was used, which led to more absorption and retention of fat in the cooked product. Ash content increased in all cooked products compared to raw samples. Ash content is an indication of the mineral content and organic constituents of food like protein, fat and fibre. The storage temperature which is suitable to increase the level of organic constituents of food, will lead to an increase in the ash content of that food. Therefore, in the current investigation, ash content was found high in T3. Compared to different cooking methods, roasted samples contained a higher amount of ash compared to deep-fried samples. This could be because roasting causes less reduction in organic constituents such as protein, fat, and fibre compared to deep frying.

The results obtained in the present study are in agreement with reports outlining that roasting increases the crude fibre and ash content in food (29). Another study observed that the high temperature used when roasting foods can increase the ash content in bar Salak (a species of palm tree). On the contrary, in deep frying, the ash content was reduced because of the reduction of fibre content and other nutrients like protein and fibre (30).

Dietary fibre is resistant to digestion by human digestive enzymes. It manages large intestine functions and has significant physiological effects on mineral bioavailability, lipid, and glucose metabolism (31). The soluble and insoluble nature of dietary fibre mainly determines its solubility in water. Soluble dietary fibre draws bodily water into the digestive tract like a magnet (32). In the gut, soluble fibre makes a gel-like structure (water soluble) and helps the food to move easily (33). Cellulose, hemicellulose, resistant starch, and lignin are the type of water-insoluble fibre, which do not dissolve in water. Insoluble fibre increases intestinal pressure, assisting in the evacuation of faeces (34). In the present study, the amount of soluble, insoluble and total dietary fibre was increased in cooked samples compared to raw samples. The increase was found to be dependent on the amount and duration of cooking temperature, as heat treatments of wheat and barley flours at 100°C convert the insoluble dietary fibre into a soluble form by increasing the water extract viscosities (35). This is the main reason for the high amount of soluble fibre content in T1 compared to T3 in the study. During thermal treatments, Maillard’s reaction increases the amount of insoluble dietary fibre content (36).

In another previous study, cooking cereals into *chapatti* had an impact on the total and insoluble dietary fibre content except from ragi, in which total dietary fibre (30 per cent) and insoluble dietary (36 per cent) increased after cooking. This increase in the total dietary fibre of ragi may be due to the formation of resistant starch (37). Hence, an increase in cellulose content led to an increased amount of TDF and IDF. Storing products at low temperatures also led to an increase in resistant starch, which is responsible for the higher amount of insoluble dietary fibre and total dietary fibre content. *In-vitro* rapidly digestible starch was reduced during boiling and frying because of a significant increase in both the resistant starch (RS) and water-insoluble dietary fibre (IDF). The total dietary fibre content increased after cooking because of the increase in cellulose, lignin and pectin content during soaking and cooking (38). In the present study, boiled *dalia* had the highest amount of total dietary fibre which may be because during boiling there is the formation of fibre-protein complexes that are resistant to heat and digestion, leading to the production of increased dietary fibre content (39). Treatment 3 led to a maximum increase in the dietary fibre content, which could be attributed to increased insoluble dietary fibre and resistant starch content.

Whole wheat flour contained 0.50 g and refined wheat flour contained 0.65 g of resistant starch, respectively, (40). Retrogradation of resistant starch rate depends on starch properties like its type of structure, cooking and storage duration, time and temperature (11). In the current investigation, we found that *dalia* followed by *parantha*, *chapatti* and *poori,* stored at 4°C for 24 h (T3) followed by stored at room temperature (T2) had higher RS content than their counterparts. The storage and reheating of products in treatment 1 (i.e., freshly prepared and treatment 4 reheating) caused a reduction of RS content due to the degeneration of the crystalline structure of starch granules as compared to T3, meaning it led to retrogradation and recrystallization of starch granules, resulting in higher RS. Compared to the food products prepared using different cooking methods, we observed that *dalia* prepared by boiling had higher RS content because, during boiling, food comes into contact with both heat and water, leading to the swelling of starch cells and its gelatinization, which results in the release of amylose in the solution (41, 42). A higher level of gelatinization occurs with an increase in the duration of cooking, leading to a higher RS content of the product.

*Chapati* prepared by roasting also had an RS content of 2.37 per 100 g. This was due to the damaging of starch cells and its partial gelatinization, which affected the formation of resistant starch, i.e., less than the boiled food product (43). Similarly, this occurred in *Parantha* (which are cooked by shallow frying) due to the formation of lipid-amylose complex. This form of RS5 led to there being a higher amount of resistant starch than *Poori*, which was prepared by deep-frying. More water was evaporated during deep-frying, which led to less formation of crystalline structure and, hence, lower resistant starch formation (25, 44). All the results revealed that the resistant starch content in wheat is inversely proportional to the non-resistant starch content and starch digested. RS is directly proportional to the total starch content, insoluble dietary fibre, and protein content (45). Hence, non-resistant starch was found high in freshly prepared and reheated wheat products compared to others. One study indicated that cereals contained less resistant starch content (25.07–31.59%) and the highest amount of non-resistant starch (30.12–56.67%) (46). The foregoing results indicated that the cooking method, like boiling and the storage temperature (stored at 4°C for 24 h), affected the formation of the RS content in wheat products by retrogradation of starch after boiling and cooling.

No significant difference was seen in the total starch content of raw and cooked samples after cooking. Only the type of starch (resistant and non-significant starch) was affected. Results showed that the total starch content in wheat products was between 70.46 to 71.5%. Freshly prepared products had the highest level of total starch content present in *Dalia* (boiling) and lowest in *chapati* (roasting). During roasting, when food came into contact with heat, the total starch was broken down into sugar. The total starch content of Pearl millet was found to be reduced after the action of the amylase enzyme when total starch was converted into sugar (47). On the contrary, the effect of different storage temperatures on total starch content indicated that freshly prepared products had high content, which decreased with increasing duration of storage. The content was reduced when exposed to high temperatures (25°C) for 6 months of storage studied (48, 49).

Plant genotype can also affect the digestibility of starch, with the amylose/amylopectin ratio being a crucial determinant of the rate of starch hydrolysis in both peas and wheat (50). The starch digestion rate was high in freshly prepared and reheated wheat products because of the presence of more non-resistant starch and less amylose content. The starch digestion rate was low in wheat products stored at 4°C and at room temperature because of retrogradation which occurs at low temperatures leading to the formation of resistant starch, which escapes digestion and absorption in the small intestine. It therefore provides satiety for a longer period of time.

The frequency and quantity of starch digestion are also affected by the structure, composition, processing, and cooking of starch granules associated with other nutrients like lipids, protein, fibre, minerals, and antinutritional factors (50, 51). Compared to different cooking methods, *dalia* prepared by boiling had the lowest starch digestion rate compared to *parantha* (shallow frying), *chapati* (roasting), and *poori* (deep frying). This variation in the digestibility of wheat products occurred due to the heat–moisture remedy proving that these treatments alter the structure of starch and increase the quantities of SDS and RS at the same time as decreasing the proportion of RDS. Differences in the physical and morphological properties of cereal starches cause lower digestibility.

Cooking cereal starches brings about modifications, for example in the physical and chemical disruption and gelatinization of starch granules. The extent of gelatinization in turn is dependent on the amount of water present, the cooking time and the temperature (52), which is the reason for the low digestibility of wheat products prepared by boiling. Studies have indicated that wheat starch may swell more slowly than other starches, which may also restrict the amount of starch gelatinization (53). The presence of protein bodies around starch granules might also limit granule swelling and starch gelatinization and as a result, reduce the susceptibility to enzymatic attack. This could be partly accountable for its low digestibility. The nature of starch also influences how it is digested. When there is a higher material content of amylose there is a decrease in the starch digestibility. Obvious variations in the digestibility of amylose and amylopectin are attributed to the larger surface area of amylopectin and the distinctly prepared and insoluble aggregates, which might lower the accessibility of cleavage sites to enzyme attack. As a result, the nature and source of starch in cereals might also have an impact on their digestibility (54).

The amylose content in raw samples increased after cooking because during cooking and cooling the amylose aligned themselves and associated with each other during retrogradation (55). *Dalia* (boiling) had a high amount of amylose content compared to *parantha* (shallow frying), *chapati* (roasting), and *poori*(deep frying) and was found to be highest at storage temperature T3 (4°C for 24 h). Wheat starches stored at T3 showed higher retrogradation rates due to longer amylopectin chains and high amylose content (56). Hence, the high amylose content is directly proportional to the high resistant starch content. Due to the presence of water in *Dalia,* the leaching out of amylose in water during boiling (57) led to a greater amount of amylose content in *dalia*, which has been linked to a greater retrogradation tendency in starches (58). Amylopectin and intermediate materials also play an important role in starch retrogradation during refrigerated storage (59). Better transition temperatures for cereal products might also result from there being a greater number of rigid granular structures and, due to the presence of lipids (60), might be the cause of resistant starch forming in *parantha*. Extra energy is needed to start melting due to the fact that amylopectin plays a chief function in starch granule crystallinity, and because amylose lowers the melting factor of crystalline regions as well as the power created by starting gelatinization in the absence of amylose-rich amorphous regions. This correlation suggests that starch with higher amylose content has extra amorphous regions and much less crystalline structures, which lower the gelatinization temperatures (61).

Amylopectin content was found to be high in freshly prepared products because after cooking and cooling (T3) the formation of amylose increased, resulting in low amylopectin content and a high amount of resistant starch. The formation of amylopectin content was inversely proportional to the amylose content. The starch from grains such as maize, wheat, rice and low amylose maize, tubers such as potatoes and sweet potatoes, and legumes such as kidney beans, has the highest starch content (8.51%), while kidney bean starch has the higher amylose content (49.50%). Large-size starches (including potato, sweet potato, and kidney bean starches) showed longer amylopectin chains high amylose content and resistant starch confirming that they had the most slowly digestible starch content (62).

The glycemic index determines the rate of rise of blood glucose by a specific food. It was found low in *dalia* compared to *chapati* when stored at 4°C (T3) compared to freshly prepared (T1). Because of the presence of retrograded starch at low temperatures. It takes time to digest and hence, slowly raises blood glucose levels. Low glycemic index diets improve both diabetic and healthy people’s risk of coronary heart disease. Low-glycemic index meals promote satiety and help limit food consumption in obese or overweight people. A healthy person’s post-prandial glucose and lipid metabolism is improved by choosing foods with a low glycemic index (63). The GL of a normal serving of food is made from the quantity of available carbohydrates and the GI of the type of food. The higher the GL, the higher the chances of elevation in blood glucose level and the insulinogenic impact of the food. The consumption of a diet with a particularly high GL for a long period of time is associated with an increased chance of type 2 diabetes and heart disease (64). Food which has a low glycemic index is also found to have a low glycemic load.

The effect of high RS foods on the blood glucose level of rats was determined in a rat trial. After consumption of *chapati* stored at T2 (room temperature for 24 h) for 28 days, it was found to be more effective in reducing blood glucose levels than consumption of products stored at T1 and T4. This happens because wheat products stored at room temperature comprise excessive quantities of nutritional fibre and resistant starch. These products release sugar slowly in the blood and also diminish glucose absorption. The gradual digestion of resistant starch (RS) has implications for the application of managed glucose release (2). The metabolism of resistant starch takes place 5–7 h after intake, compared to starch that is cooked using more conventional approaches. As previously mentioned, digestion takes 5-7 h, and slowly increases blood glucose levels resulting in reduced postprandial glycaemia and insulinemia and providing satiety for longer (65). The effect of insoluble nutritional fibre in the inhibition of glucose diffusion in the small gut is usually recommended because of the absorption or inclusion of smaller sugar molecules (66). In the current investigation, we observed that GLP-1 and peptide YY production were increased by the consumption of products containing resistant starch and dietary fibre. This stimulates insulin secretion and reduces glucagon secretion (67). *In vivo* research showed that fibre and resistant starch in the digestive system of rats act as the main factors that slow glucose absorption and reduce the rise of blood glucose levels by promoting glycogen synthesis (68) and inhibition of gluconeogenesis (69).

## 5 Conclusion

Cooking methods including boiling, roasting and shallow frying increased while deep frying decreased the amount of resistant starch in foods. Products stored at 4°C (T3) and at room temperature for 24 h (T2) showed an increased amount of resistant starch whereas freshly cooking (T1) and reheating (T4) decreased the amount of resistant starch of foods. Products stored at 4°C (T3) have a high amount of insoluble dietary fibre, slowly digestible starch and amylose content. The glycemic index and glycemic load were also found to be low in T3 wheat products. These contained a high amount of resistant starch, which reduced the blood glucose level by regulating the promotion of glycogen synthesis and inhibition of gluconeogenesis. Consumption of *chapati* (T3) in a rat study was found to be more beneficial in controlling the rise of blood glucose levels. Thus, it leads to a slower rise in blood glucose levels, providing longer satiety. In India, people consume a large variety of starchy preparations, meaning that modifications to the food cooking methods and storage temperatures used for starchy foods may lead to several health benefits. Greater awareness should be fostered regarding the nutritional and health benefits of resistant starch consumption to maintain blood levels. People also need to be educated about the correct ways of cooking and storing food products to increase the amount of resistant starch in food at the domestic level.

## Data availability statement

The original contributions presented in the study are included in the article/Supplementary material, further inquiries can be directed to the corresponding author/s.

## Ethics statement

The studies involving humans were approved by the Institutional ethics committee, PAU. The studies were conducted in accordance with the local legislation and institutional requirements. Written informed consent for participation was not required from the participants or the participants’ legal guardians/next of kin because there was no chance to harm the human. In the study only their blood glucose levels was measured. Written informed consent was obtained from the individual(s) for the publication of any potentially identifiable images or data included in this article.

## Author contributions

PK: Writing – original draft. HK: Methodology, Writing – review & editing. RA: Writing – review & editing. KB: Visualization, Writing – review & editing. AM: Data curation, Writing – review & editing. OG: Project administration, Writing – review & editing. LS: Visualization, Writing – review & editing. KS: Visualization, Writing – review & editing.

## References

[1] Englyst HN Kingman SM Cummings JH. Classification and measurement of nutritionally important starch fractions. Eur J Clin Nutr. (1992) 46:33–50. 1330528

[2] Nugent AP. Health properties of resistant starch. Nutr Bull. (2005) 30:27–54. doi: 10.1111/j.1467-3010.2005.00481.x

[3] Patil SK. Resistant starches as low-carb ingredients - current applications and issues. Cereal Foods World. (2004) 49:292–4.

[4] Tapsell LC. Diet and metabolic syndrome: where does resistant starch fit in? J Assoc Official Analyt Chem. (2004) 87:756–60. doi: 10.1093/jaoac/87.3.756 15287676

[5] Murphy MM Douglass JS Birkett A. Resistant starch intakes in the United States. J American Dietetic Assoc. (2008) 108:67–78. doi: 10.1016/j.jada.2007.10.012 18155991

[6] Raigond P Ezekiel R Raigond B. Resistant starch in food: a review. J Sci Food Agric. (2015) 95:1968–78. doi: 10.1002/jsfa.6966 25331334

[7] Sajilata MG Singhal RS Kulkarni PR. Resistant starch–a review. Compr Rev Food Sci Food Saf. (2006) 5:1–17. doi: 10.1111/j.1541-4337.2006.tb00076.x 33412740

[8] Hernández O Emaldi U Tovar J. In vitro digestibility of edible films from various starch sources. Carboh Polym. (2008) 71:648–55. doi: 10.1016/j.carbpol.2007.07.016

[9] Sanz-Penella JM Tamayo-Ramos JA Sanz J Haros M. Phytate reduction in bran-enriched bread by phytase producing bifidobacteria. J Agric Food Chem. (2009) 57:10239–44. doi: 10.1021/jf9023678, PMID: 19817458

[10] Fuentes-Zaragoza E Sánchez-Zapata E Sendra E Sayas E Navarro C Fernández-López J. Resistant starch as prebiotic: a review. Starch - Stärke. (2011) 63:406–15. doi: 10.1002/star.201000099

[11] Sakac N Karnaš DJ Jozanović HM Gvozdić V Kovač-Andrić E Sakač M. Application of spectrophotometric fingerprint in cluster analysis for starch origin determination. Food Technol Biotechnol. (2020) 58:5–11. doi: 10.17113/ftb.58.01.20.6239 32684782 PMC7365342

[12] Chung H Hoover R Liu Q. The impact of single and dual hydrothermal modifications on the molecular structure and physicochemical properties of normal corn starch. Int J of Biolo Macro. (2009) 44:203–10. doi: 10.1016/j.ijbiomac.2008.12.007, PMID: 19136026

[13] Ozturk S Koksel H Perry KWN. Characterization of resistant starch samples prepared from two highAmylose maize starches through debranching and heat treatments. Cereal Chem. (2011) 86:503–10. doi: 10.1094/CCHEM-86-5-0503

[14] Margareta Leeman A Karlsson ME Eliasson AC Bjorck IME. Resistant starch formation in temperature treated potato starches varying in amylose/ amylopectin ratio. Carbohydr Polym. (2006) 65:306–13. doi: 10.1016/j.carbpol.2006.01.019

[15] Muir JG O'dea K Cameron-Smith D Brown YL Collier GR. Resistant starch-the neglected dietary fiber? Implication for health. A review of the effects of dietary fiber and resistant starch on small and large bowel physiology. Dietary Fibre. (1993) 1:33–47.

[16] Reader D Johnson ML Hollander P Franz M. Response of resistant starch in a food bar vs. two commercially available bars in persons with type II diabetes mellitus. Diabetes. (1997) 46:254.

[17] Cummings JH Englyst HN. Measurement of starch fermentation in the human large intestine. Can J Physiol Pharmacol. (1991) 69:121–9. doi: 10.1139/y91-018 2036594

[18] Brand-Miller J Hayne S Petocz P Colagiuri S. Low-glycemic index diets in the management of diabetes: a meta-analysis of randomized controlled trials. Diabetes Care. (2003) 26:2261–7. doi: 10.2337/diacare.26.8.2261 12882846

[19] Levrat MA Moundras C Younes H Morand C Demigne C Remesy C. Effectiveness of resistant starch, compared to guar gum, in depressing plasma cholesterol and enhancing fecal steroid excretion. Lipids. (1996) 31:1069–75. doi: 10.1007/BF02522464, PMID: 8898306

[20] Higgins JA Higbee DR Donahoo WT Brown IL Bell ML Bessesen DH. Resistant starch consumption promotes lipid oxidation. Nutr Meta. (2004) 1:8. doi: 10.1186/1743-7075-1-8 PMC526391 15507129

[21] AOAC. Official methods of analysis. 17th ed. Washington, DC: Association of Official Analytical Chemists (2000).

[22] AOAC. Official methods of analysis. 16th ed. Washington, DC: Association of Official Analytical Chemists (2002).

[23] Sopade PA Gidley MJ. A rapid in-vitro digestibility assay based on glucometry for. Investigating the kinetics of starch digestion. Starch-Stärke. (2009) 61:245–55. doi: 10.1002/star.200800102

[24] Juliano BO Perez CM Blakeney AB Castillo T Kongseree N Laignelet B. International cooperative testing on the amylose content of milled Rice. Starch Stärke. (1981) 33:157–62. doi: 10.1002/star.19810330504

[25] Goni I Garia-Alonso A Saura-Calixto F. A starch hydrolysis procedure to estimate glycemic index. Nutr Res. (1997) 17:427–37. doi: 10.1016/S0271-5317(97)00010-9

[26] McLean LA Sosulski FW Youngs CG. Effects of nitrogen and moisture on yield and protein in field peas. Can J Plant Sci. (1974) 54:301–5. doi: 10.4141/cjps74-047

[27] Kibar H Sonmez F Temel S. Effect of storage conditions on nutritional quality and color characteristics of quinoa varieties. J Stored Prod Res. (2021) 91:101761. doi: 10.1016/j.jspr.2020.101761

[28] Zhang K Shi Y Zeng J Gao H Wang M. Effect of frozen storage temperature on the protein properties of steamed bread. Food. Sci Technol. (2022) 42:42. doi: 10.1590/fst.68622

[29] Adegunwa MO Adebowale AA Solano EO. Effect of thermal processing on the biochemical composition, antinutritional factors and functional properties of beniseed flour. Am J Biochem Mol Biol. (2012) 2:175–82. doi: 10.3923/ajbmb.2012.175.182

[30] Siregar NS Julianti E Silalahi J. The effect of roasting temperature on proximate and dietary fiber of food bar salak (Sidimpuan cultivar) fruit. E3S Web Conf. (2021) 332:03001. doi: 10.1051/e3sconf/202133203001

[31] Ötles S Ozgoz S. Health effects of dietary fiber. Acta Sci PolonorTechnol Aliment. (2014) 13:191–202. doi: 10.17306/J.AFS.2014.2.8 24876314

[32] Everything Kate F. You need to know about soluble Fiber (2020). Available at: http://www.katefarms.com.

[33] Cheng L Zhang X Hong Y Li Z Li C Gu Z. Characterisation of physicochemical and functional properties of soluble dietary fibre from potato pulp obtained by enzyme-assisted extraction. Int J Bio Macromol. (2017) 101:1004–11. doi: 10.1016/j.ijbiomac.2017.03.156, PMID: 28373048

[34] Akinola OO Raji TJ Olawoye B. Lignocellulose, dietary fibre, inulin and their potential application in food. Heliyon. (2022) 29:104–59. doi: 10.1016/j.heliyon.2022.e10459 PMC9449745 36090233

[35] Caprita A Caprita R Simulescu VO Drehe RM. The effect of temperature on soluble dietary fiber fraction in cereals. J Agro aliment Processes Technol. (2011) 17:214–7.

[36] Chang MC Morris WC. Effect of heat treatments on chemical analysis of dietary fiber. J Food Sci. (1990) 55:1647–50. doi: 10.1111/j.1365-2621.1990.tb03591.x

[37] Ramulu O Rao U. Effects of processing on dietary fiber content of cereals and pulses. Plant Foods Hum Nutr. (1997) 50:249–57. doi: 10.1007/BF02436061, PMID: 9373875

[38] Vasishtha Hina Srivastava RP. Dietary fiber, protein and lectin contents of lentils (*Lens culinaris*) with soaking and cooking. Current Adv Agri Sci. (2013) 5:238–41.

[39] Caprez A Arrigoni E Amado R Neucom H. Influence of different types of thermal treatment on the chemical composition and physical properties of wheat bran. J Cereal Sci. (1986) 4:233–9. doi: 10.1016/S0733-5210(86)80025-X

[40] Nigudkar MR. Estimation of resistant starch content of selected routinely consumed indian food preparations. Curr Res Nutr Food Sci. (2014) 2:73–83. doi: 10.12944/CRNFSJ.2.2.03

[41] Ek KL Brand-Miller J Copelan L. Glycemic effect of potatoes. Food Chem. (2012) 133:1230–40. doi: 10.1016/j.foodchem.2011.09.004

[42] Kolaric L Minarovičová L Lauková M Karovičováand J Kohajdová Z. Pasta enriched with sweet potato starch: impact on quality parameters and resistant starch content. J Text Stud. (2020) 51:464–74. doi: 10.1111/jtxs.12489, PMID: 31603534

[43] Kumar and Prasad. Changes in the characteristics of indica rice in the process of flaking. J Food Eng. (2018) 6:2310–7.

[44] Mangala SL Vidyasankar K Tharanathan RN. Resistant starch from processed cereals. The influence of amylopectin and non-carbohydrate constituents on its. Formation. Food Chem. (1999) 64:391–6. doi: 10.1016/S0308-8146(98)00142-3

[45] Gopinath V Saravanan S Al-Maleki AR Ramesh M Vadivelu J. A review of natural polysaccharides for drug delivery applications: special focus on cellulose, starch and glycogen. Biomed Pharmacother. (2010) 107:96–108. doi: 10.1016/j.biopha.2018.07.136 30086465

[46] Shewry PR Hey SJ. The contribution of wheat to human diet and health. Food Energy Secur. (2015) 4:178–202. doi: 10.1002/fes3.64, PMID: 27610232 PMC4998136

[47] Sharma A Kapoor AC. Effect of processing on the nutritional quality of pearl millet. J Food Sci Technol. (1997) 34:50–3.

[48] Rehman ZU Habib S Zafar SI. Nutritional changes in maize (*Zea mays*) during storage at three temperatures. Food Chem. (2002) 77:197–201. doi: 10.1016/S0308-8146(01)00337-5

[49] Liu K Liu Q. Enzymatic determination of total starch and degree of starch gelatinization in various products. Food Hydrocoll. (2020) 103:105639. doi: 10.1016/j.foodhyd.2019.105639

[50] Regmi PR Metzler-Zebeli BU Ganzle MG Van Kempen TATG Zijlstra R. Starch with high amylose content and low in vitro digestibility increases intestinal nutrient flow and microbial fermentation and selectively promotes Bifidobacteria in pigs. J Nutr. (2011) 141:1273–80. doi: 10.3945/jn.111.140509, PMID: 21628635

[51] Al-Rabadi GJS Gilbert RG Gidley MJ. Effect of particle size on kinetics of starch digestion in milled barley and sorghum grains by porcine alpha-amylase. J Cereal Sci. (2009) 50:198–204. doi: 10.1016/j.jcs.2009.05.001

[52] Williams MR Bowler P. Starch gelatinization – a morphological study of Triticeae and other starches. Starch Stärke. (1982) 34:221–3. doi: 10.1002/star.19820340703

[53] Davis EA. Wheat Starch. Cereals Food World. (1994) 36:34–9. doi: 10.1080/10261133.1994.9673935

[54] Goddard MS Young G Marcus R. The effect of amylase content on insulin and glucose responses to ingested rice. Am J Clin Nutr. (1984) 39:388–92. doi: 10.1093/ajcn/39.3.388 6364775

[55] Sagum R Arcot J. Effect of domestic processing methods on the starch, non starch polysaccharides and in vitro starch and protein digestibility of three varieties of rice with varying levels of amylose. Food Chem. (2000) 70:107–11. doi: 10.1016/S0308-8146(00)00041-8

[56] Singh N Kaur A Shevkani K. Maize: grain structure, composition, milling, and starch characteristics nutrition dynamics and novel uses Springer India (2014).

[57] Chung H Liu Q Hoover R. Impact of annealing and heat-moisture treatment on rapidly digestible, slowly digestible and resistant starch levels in native and gelatinized corn, pea and lentil starches. Carbohydr Polym. (2011) 75:436–47. doi: 10.1016/j.carbpol.2008.08.006

[58] Whistler RL BeMiller JN. Carbohydrate chemistry for food scientists. Starch. (1996):117–51.

[59] Yamin FF Lee M Pollak LM White PJ. Thermal properties of starch in corn variants isolated after chemical mutagenesis of inbred line B73. Cereal Chem. (1999) 76:175–81. doi: 10.1094/CCHEM.1999.76.2.175

[60] Singh J Singh N. Studies on the morphological and rheological properties of granular cold water soluble corn and potato starches. Food Hydrocoll. (2003) 17:63–72. doi: 10.1016/S0268-005X(02)00036-X

[61] Sasaki T Yasui T Matsuki J. Effect of amylose content on gelatinization, retrogradation and pasting properties of starches from waxy and non-waxy wheat and their F1 seeds. Cereal Chem. (2000) 77:58–63. doi: 10.1094/CCHEM.2000.77.1.58

[62] Bajaj R Singh N Kaur A Inouchi N. Structural, morphological, functional and digestibility properties of starches from cereals, tubers and legumes: a comparative study. J Food Sci Technol. (2018) 55:3799–808. doi: 10.1007/s13197-018-3342-4, PMID: 30150840 PMC6098761

[63] Rizkalla SW Bellisle F Slama G. Health benefits of low glycemic index foods, such as pulses, in diabetic patients and health individuals. BrJ Nutr. (2002) 88:255–62. doi: 10.1079/BJN2002715, PMID: 12498625

[64] Liu Q Zhang Y Laskowski JS. The adsorption of polysaccharides onto mineral surfaces: an acid/base interaction. Int J Min Process. (2000) 60:229–45. doi: 10.1016/S0301-7516(00)00018-1

[65] Raben A Tagliabue A Christensen N Madsen J Holst J Astrup A. Resistant starch: the effect on postprandial glycemia, hormonal response, and satiety. Am J Clin Nutr. (1994) 60:544–51. doi: 10.1093/ajcn/60.4.544, PMID: 8092089

[66] Lopez HW Levrat-Verny MA Coudray C Besson C Krespine V Messager A. Class 2 resistant starches lower plasma and liver lipids and improve mineral retention in rats. J Nutr. (2001) 131:1283–9. doi: 10.1093/jn/131.4.1283, PMID: 11285339

[67] Zhou J Matrin RJ Tulley RT Raggio AM McCutcheon KL Shen L. Dietary resistant starch upregulates total GLP-1 and PYY in a sustained day-long manner through fermentation in rodents. Am J Physiol Endocrinol Metab. (2008) 295:E1160–6. doi: 10.1152/ajpendo.90637.2008 18796545 PMC2584810

[68] Ou S Kwok KC Li Y Fu L. In vitro study of possible role of dietary fiber in lowering postprandial serum glucose. J Agric Food Chem. (2011) 49:1026–9. doi: 10.1021/jf000574n 11262066

[69] Wood L Wilbourne J Kyne-Grzebalski D. Administration of insulin by injection. Pract Diabet Int. (2002) 19:S1–4. doi: 10.1002/pdi.330

